# Immunogenicity evaluation after BNT162b2 booster vaccination in healthcare workers

**DOI:** 10.1038/s41598-022-16759-2

**Published:** 2022-07-26

**Authors:** Sabina Zurac, Cristian Vladan, Octavian Dinca, Carolina Constantin, Monica Neagu

**Affiliations:** 1 Faculty of Dental Medicine, “Carol Davila” University of Medicine and Pharmacy, Bucharest, Romania; 2 Department of Pathology, Colentina Clinical Hospital, Bucharest, Romania; 3 “Prof. Dr. Dan Theodorescu” Clinical Hospital for Oro-Maxillo-Facial Surgery, Bucharest, Romania; 4 Department of Immunology, Victor Babes National Institute of Pathology, Bucharest, Romania; 5 Doctoral School, Faculty of Biology, University of Bucharest, Bucharest, Romania

**Keywords:** Cell biology, Immunology, Diseases, Health care

## Abstract

Waning of the immune response upon vaccination in SARS-CoV-2 infection is an important subject of evaluation in this pandemic, mostly in healthcare workers (HCW) that are constantly in contact with infected samples and patients. Therefore, our study aimed to establish the specific humoral response of specific IgG and IgA antibodies upon vaccination, during the second year of pandemic and evaluating the booster shot with the same vaccine type. A group of 103 HCW with documented exposure to the virus were monitored for specific IgG and IgA levels prior to vaccination, after the first vaccination round, during the following 8 months and after the booster shot with the same vaccine type. After 8 months post-vaccination the humoral response in both IgG and IgA decreased, 2.4 times for IgG, and 2.7 times for IgA. Although the antibodies levels significantly decreased, no documented infection was registered in the group. After the booster shot, the entire group, displayed IgG increased levels, immediately after booster followed by the increase in specific IgA. IgG levels post-second round of vaccination are statistically higher compared to the first round, while IgA is restored at the same levels. Within the vaccination or booster routine for a multiple waves’ pandemic that is generating new virus variants, populational immunity remains an important issue for future implementation of prevention/control measures.

## Introduction

Entering the third year of COVID-19 pandemic and registering in Romania already over 2.6 million cases^1^ with over 62,000 deaths^2^ the vaccination route of the population reached just a 36% percentage of the total population. As COVID-19 will enter its endemic phase, prevention and control raise severe challenges. In the third year of COVID-19 we still have no specific treatment, and although face masks wearing, social distancing and cautious hands hygiene represent important measures for controlling COVID-19 pandemic spreading^3^, promoting vaccinations and developing herd immunity are the only effective and economic measures to control the current pandemic^4^.

Extended studies that focus on the antibody levels triggered by infection and/or by vaccination have reported the existance of an entire panel of specific immunoglobulines^5^. Moreover, recent studies show that cross immunity against coronaviruses can be elicited by vaccination^6^ but still we have to focus on the relevance of the booster vaccination. Within the total population, healthcare workers (HCW) represent the highly exposed populational segment to virus threatening. Nevertheless, since the beginning of the global vaccination campaign, many studies have referred to the effectiveness of this active immunization against SARS-CoV-2. A recent real-world setting meta-analysis related to the vaccination effectiveness in fully vaccinated peoples has included multiple records from worldwide. Thus, in those 51 records the vaccination output was assessed in relation with infectivity, hospitalization, ICU admission and COVID-19 associated death, proving efficacy in young adults (86.1%), elderly 83.8% and HCW (95.3%)^7^. Furthermore, a preliminary investigation of the vaccine effectiveness in Romania run in February–May 2021 revealed that full-scheme vaccination decreases the risk of infection by 10 times while the risk of hospitalization and ICU admission is 12 times lower; moreover, the risk of decease from COVID-19 is reduced by 14 times^8^. In Romania the rate of populational vaccination was reported as 42.8%, while HCW had an overall vaccination rate of 70%^9^.

Therefore, monitoring HCW characteristics and response to vaccination represents a good overall example of vaccine efficacy. Moreover, evaluating vaccine efficacy against SARS-CoV-2 variants is seminal to sustain proper information to the large population and to guide public health in this pandemic^10^. A recent finding suggests that the mRNA vaccine booster, associates with a good protection against Omicron and Delta variants when comparing the effect to unvaccinated or to the two doses vaccination^11^. In an Italian cohort comprising almost 1 year of follow-up and over 33 million tested subjects important issues emerged. When epidemic phase registered Delta variant circulation vaccine effectiveness decreased from 82 to 33% at 7 months after the second dose. Moreover, the study showed that high risk individuals aged ≥ 80 years after 7 months seemed not to be protected after the second dose of vaccine. Therefore, the authors sustain a booster vaccination even earlier than 6 months after the primary vaccination cycle^12^. The Israeli reports done on immunity wanning and booster reccomandation are numerous. Thus, in August 2021, in Israeli HCW, the surge of SARS-CoV-2 infections, mostly by Delta variant, appeared in 21.4% individuals that received only the two-dose regimen while the rate in the HCW group that have received a booster was only 0.7%. Therefore, in this group, a booster vaccination indicates substantial protection by a third vaccine dose^13^ while previous studies in the same country have shown that at 3 months most HCWs still had measurable antibodies^14^. Nevertheless, in the same country at 5 months, a third dose of the BNT162b2 mRNA vaccine is effective in protecting subjects against severe COVID-19, compared with the subjects receiving only two doses^15^. Additionally, half a year after first vaccination with the BNT162b2 vaccine second dose, the humoral response was found substantially decreased, more specifically in men, over 65 years of age or older, and among immunosuppressed subjects^16^.

When examinating total and neutralizing antibodies raised in HCW against SARS-CoV-2 Spike protein, from Washington-1 (WA-1), Beta, Delta and Omicron variants of concern it was shown that mRNA booster eliminates the immune escape phenomena observed with the Omicron variant after two-dose vaccination^17^. Another study has shown that although neutralizing antibodies raised by two-dose vaccination decreased 5 months after the second vaccination, specific T and B lymphocytes were still detectable, and upon 3rd dose induced a quick recall response. An interesting finding of the study showed that although HCWs with low antibodies response to two doses prooved good specific immune memory, that was quickly recalled by the third dose^18^. In over 3000 HCW subjects from an Italian hospital, infection after vaccination occurred in 0.5% subjects mostly asymptomatic with no predominance of a specific viral variant^19^. Somewhat similar results were obtained in a Turkish HCW cohort were 4.5% of vaccinated personel were infected with SARS-CoV-2^20^ and the booster dose of CoronaVac was advised^21^.

Combination of vaccination has shown that combining mRNA-mRNA or vector-mRNA types induces high neutralization titers against SARS-CoV-2^22^. Another combination study done in Spanish HCW has reported results for the combination of one dose of ChAdOx1-S-nCoV-19 followed by a second dose of the Pfizer BNT162b2 vaccine as a booster. The heterologous vaccinated subjects proved a stronger neutralizing activity no matter of the SARS-CoV-2 variant. The enhanced neutralizing potential is due to the appereance of switched and activated memory B cells^23^. A study published almost concomitantly with the later one, has shown that T cell activation markers increase after vaccination. Plasma from previously infected subjects or 3 dose vaccinated subjects had a better neutralization capacity compared to the plasma harvested from non-infected individuals receiving two vaccine doses^24^.

In CoronaVac vaccination it was shown that after 6 months post-vaccination almost all HCW subjects has prooved a decreased antibody persistence^25^. AZD1222 (ChAdOx1) vaccination study has shown also an important decline in antibody levels in HCW, months after vaccination^26^. In a Korean HCW BNT162b2 vaccinated cohort it was shown that after 6 months, serum antibody levels significantly declined^27^. In Finland, mRNA vaccine displayed only 53% from the initial IgG level after 6 months, but antibody waning was not observed against COVID-19 hospitalization^28^. In a HCW Polish cohort it was reported that there are higher levels of specific antibodies 6 months after vaccination in subjects experiencing the disease after the first round of vaccination, the finding supporting once more the use of a booster dose, especially for non-infected subjects^29^.

In Indonesian HCW specific IgG persisted 3 months post-vaccination with an inactivated SARS-CoV-2 vaccine. The authors point out that there is an increased decline of the specific antibodies in subjects without prior SARS-CoV-2 infection, finding that sustains the need for an additional booster dose^30^.

IgA is an antibody that sustains the humoral mucosal immunity especially in viral respiratory infections, and that there are few studies that evaluate the circulatory form of the antibody in COVID-19^31,32^. We have previously shown that post-vaccination, specific serum IgA is triggered in similar levels with IgG and having the same antibody dynamics^33^, while other studies have reported saliva IgA in low levels upon vaccination^34^. At 6 months, post-vaccination specific IgA serum levels showed a significant descending trend^35^. In a Dutch cohort vaccination with several vaccine types (mRNA-1273, BNT162b2, Ad26.CoV2-S or ChAdOx1-S) was studied and the authors point out that specific T cell responses were detectable 1-year post-vaccination while the humoral responses retained up to 4 months^36^.

Immune response wanning upon vaccination in COVID-19 is an important issue in the current pandemics, mostly in HCW. Therefore, our study aimed to establish the specific humoral response of antibodies IgG and IgA, upon specific vaccination, during the second year of pandemia and evaluating the booster shot with the same vaccine type and dose.

## Materials and methods

### Subjects

A total of 103 subjects, HCW in contact with SARS-CoV-2-infected samples and patients, constituted the test group followed-up between May, 2020 and October, 2021. The entire HCW group was involved in tertiary care and in contact only with COVID-19 patients. Hence, HCW had direct exposure to infected patients, infected samples and patients dead due or with SARS-CoV2 virus. The characteristics of the enrolled subjects, such as age and sex are presented in Table 1 along with their associated co-morbidities.

**Table 1:** Characteristics of the tested subjects.

Parameter	Infected subjects until January 2021 (%)	Non-infected subjects until January 2021 (%)	Infected subjects during January–October 2021 (%)
Subjects (n)			
Female (90)	23	77	0
Male (13)	29	71	0
Average age of total (years)	37.81	41.00	40.26
Average age of women (years)	39.14	41.48	40.95
Average age of men (years)	28.50	36.40	34.14
Major comorbidities (%)	Overweight (BMI ≤ 25)		23
	Non-obesity overweight (BMI = 26–30)		13
	Obesity (BMI > 30)		10
	Cardiovascular disease		9
	Arterial hypertension		6
	Diabetes		4
	Non-immune thyroidian disease		4
	Hypothyroidism		4
	Autoimmune thyroiditis		4
	Allergies		3
	Chronic venous insufficiency		3
	Various other comorbidities		Under 2%

The group of 103 subjects were vaccinated in January, 2021 and they were followed-up before and after vaccination for measurement of the levels of serum IgG and IgA, during the 8 months of surveillance, prior to the 3rd booster received in October 2021 and after 3 weeks post-booster. Monthly RT-PCR tests during the 8 months follow-up yielded negative results for all subjects.

### Vaccination

All the subjects received the Pfizer-BioNTech vaccine according to the supplier instructions, namely they received their first vaccine shot on the January 6, 2021 and the second dose on January, 27 and the results of a sample of the tested group were prior published by us focusing on the humoral response triggered by the first vaccination protocol^33^. Subjects were followed the entire 2021 year and in October 2021 they have received the booster shot with the same Pfizer-BioNTech vaccine. All experiments were performed in accordance with relevant guidelines and regulations in accordance with the Declaration of Helsinki. All enrolled subjects signed an informed consent and the study was approved by the Ethics Committee from Colentina Hospital (25/2017). All methods were carried out in accordance with guidelines and regulations.

### Dynamics of sampling

All the subjects were tested for the presence of IgA and IgG-specific antibodies recognizing the S1 domain of the SARS-CoV-2 Spike protein. Pfizer-BioNTech vaccination scheme comprised a first shot followed by a 21 days booster in January 2021. All subjects were tested 1 day prior to Pfizer-BioNTech vaccination, after 2 weeks post completion of the vaccination scheme), 1 day before the 3rd booster (8 months after the first vaccination scheme) and 3 weeks post-booster. Within the entire group, 15 subjects agreed to be tested weekly and results for one subject are presented hereafter.

### Blood sampling

Peripheral blood samples from subjects were collected by venipuncture during the morning hours in blood clot activator tubes (Vacutest Kima). Blood collection was carried out at the Colentina Clinical Hospital. Serum samples, separated by centrifugation (1500 × *g*, 10 min at room temperature) within 4 h of blood collection, were used for ELISA. Serum samples were stored at − 80 °C for concomitant testing.

### ELISA

Anti-SARS-CoV-2 ELISA (IgG and IgA) was used to determine the serum levels of specific IgG and IgA (EUROIMMUN Medizinische Labordiagnostika AG). The used protocol was as per the manufacturer’s instructions. Details of the standard ELISA test were prior presented by us^33^. Results were calculated as indicated, namely the Ratio between the *Extinction of the patient sample* and the *Extinction of the calibrator*. The manufacturer recommends the following cut-off values: Ratio < 0.8; Borderline Ratio ≥ 0.8 to < 1.1; Positive Ratio ≥ 1.1.

The results are presented as index, as recommended by the IgG/IgA kit supplier. Data are presented as the mean ± standard deviation (SD). Comparison between groups, data analysis was performed using One-way ANOVA or Mann–Whitney tests using GraphPad Prism 9.31 (471) (GraphPad Software, Inc. www.graphpad.com).

All the tests and methods were performed in accordance with the relevant guidelines and regulations.

### Informed consent

All subjects signed an informed consent and the study was approved by the Ethics Committee from Colentina Hospital (25/2017).

## Results

### Demographics characteristics

The tested group consists of mainly females having a mean age of 40.26 years with various comorbidities as presented in Table 1. Allergies that were registered in the HCW group are toward drugs, various food components and atopic dermatitis to metals. The presented autoimmune thyroiditis is an autoimmune disease, hypothyroidism is characterized by an underactive thyroid producing fewer thyroid hormones while non-immune thyroidian disease reflects the dysfunction of the thyroid gland mainly hyperthyroidism.

As previously reported by us, the gender differences did not statistically influence the level of antibody response upon vaccination, therefore the presented results comprise the entire group regardless of the gender.

### Dynamics of IgG and IgA antibodies

**Figure 1:**
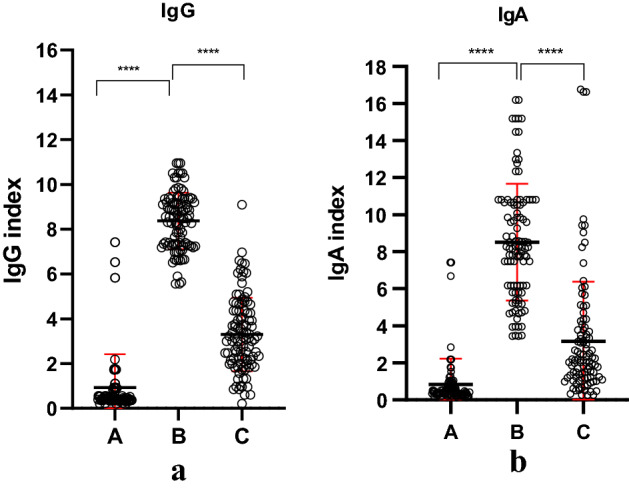
Ig indexes dynamics for the entire group regardless of their prior infection or not, before vaccination (A), after completion of the vaccination scheme (B) and 8 months after vaccination (C). (**a**) IgG index; (**b**) IgA index (red line mean ± SD). Figure was done using GraphPad Prism 9.31 (471) (GraphPad Software, Inc. www.graphpad.com).

The group received the Pfizer-BioNTech vaccination scheme in January 2021. Regardless of the infection status prior to vaccination, the entire group presented a high IgG and IgA levels post first round of vaccination (Fig. 1).

After 8 month post first round of vaccination, the group had statistically decreased values for both antibodies (Fig. 1). More specifically, for IgG the mean concentration decreased in 8 month 2.4 times, while for IgA decreased 2.7 times (p < 0.001). To be mentioned that in the 8 months time period (January–October 2021) no documented infection with SARS-CoV-2 was registered.

**Figure 2:**
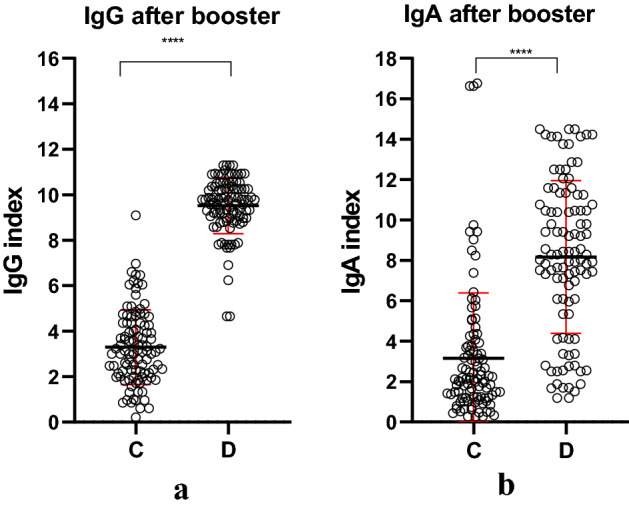
Ig indexes dynamics for the entire group regardless of their prior infection or not, 8 months after vaccination (C) and after booster vaccination (D); (**a**) IgG index; (**b**) IgA index (red line mean ± SD). Figure was done using GraphPad Prism 9.31 (471) (GraphPad Software, Inc. www.graphpad.com).

The entire group was subjected to booster vaccination in October 2021 and post secound round of vaccination the immunoglobulins serum concentrations (Fig. 2) show that IgG increases imediately after booster 2.7 times, while IgA increased after the booster 2.5 times (p < 0.001).

**Figure 3:**
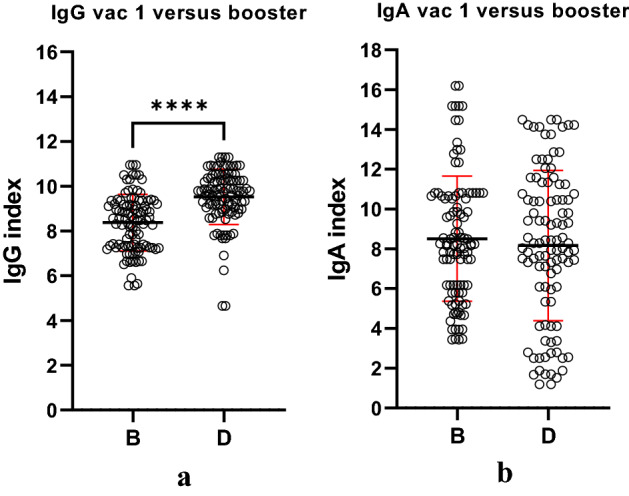
Ig indexes dynamics for the entire group regardless of their prior infection or not, first vaccination (B) compared to booster vaccination (D); (**a**) IgG index; (**b**) IgA index (red line mean ± SD). Figure was done using GraphPad Prism 9.31 (471) (GraphPad Software, Inc. www.graphpad.com).

To evaluate the level of humoral induction after booster vaccination we have compared, yet again the entire group with the values obtained after the first vaccination (Fig. 3). Results shown that the IgG response after booster vaccination is statistically higher compared to the one obtained by the first vaccination (p < 0.001). In contrast, the IgA response after booster is almost identical to the values obtained after the first vaccination.

**Figure 4:**
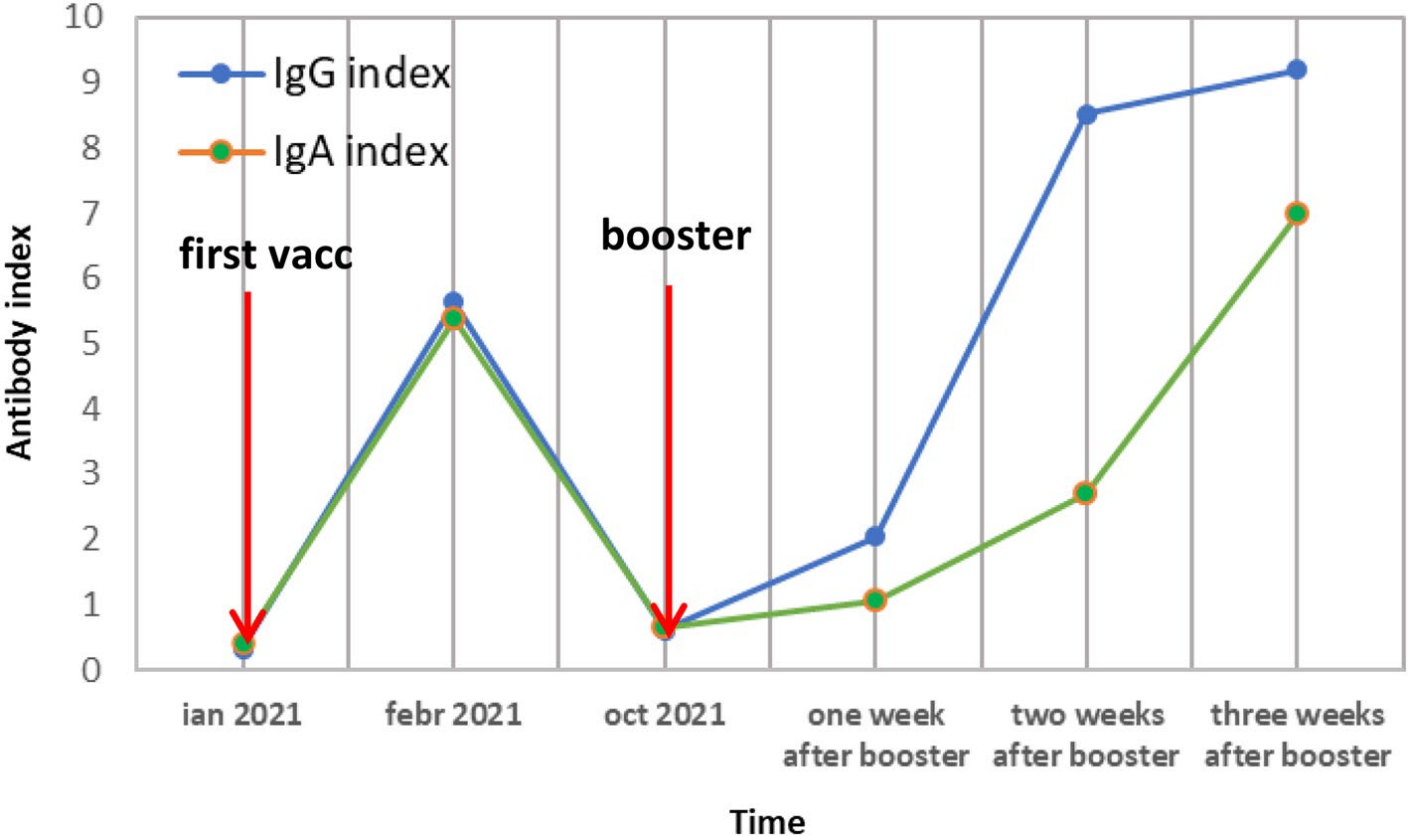
Individual values of IgG and IgA indexes after the first vaccination and booster in a non-infected individual. Figure was done using GraphPad Prism 9.31 (471) (GraphPad Software, Inc. www.graphpad.com).

Some of the enrolled subjects were tested in a more detailed dynamics to evaluate the time frame in which the humoral response appears after the booster vaccination. Thus, in a case where after 8 months post-first vaccination there are no detectable circulating antibodies, the booster induced a rapid (after the first week post-booster) a high value for both IgG and IgA, the levels continued to raise after two, respectively 3 weeks after booster. The concentrations of serum IgG and IgA were continuing to increase 1 month after booster (Fig. 4). Both registered values were in this case higher than the ones registered post first-vaccination prooving a proper immunological memory. However, 6 months after booster (174 days), the subject developed a mildly sympthomatic form of COVID-19 documented by positive RT-PCR test, Omicron variant sequencuing preceded by the rapid antigen test. Symptomatology was associated with Omicron variant infection (sore throat, rinorhea, cough and harsh voice for 2 days, no fever, no headache, no other symptoms; oxygen saturation 96–100) having family members tested negative by rapid antigen tests.

### Respiratory infections prior to booster vaccination

Out of the entire study group, during the 8 months follow up after first vaccination none of the subjects have contracted the SARS-CoV-2 infection pre-3rd vaccination booster. The assertion refers to the lack of any symptomatology related to the respiratory infection and to the fact that the in routine check-ups using RT-PCR testing no positive results were documented in this time frame.

### Adverse effects upon vaccination

The presence of any adverse effects for the 3rd booster were registered for each subject. The adverse effects registered were compared to the ones registered in the first scheme of vaccination and in the entire group the adverse effects reported by us for the same group after the first round of vaccination^33^ were less intense and far more reduced in number. Similar to the first round of vaccination, booster induced milder injection site pain in over 75% of the subjects.

### Specific antibodies level upon 3rd vaccination

In the presented study, as the group displayed a decrease in both IgG and IgA increment of specific antibodies, the booster shot re-established, and for IgG even increased the specific humoral response in all of subjects. An interesting finding was that after booster vaccination the newly achieved level of IgA was statistically identical to the one achived after the first scheme of vaccination, while specific IgG surpassed the prior achieved antibody levels, sustaining the existance of a robust cellular memory.

### COVID-19 after booster

9 subjects from our study group (8.73%) developed COVID-19 after booster. One of the subjects developed the infection with Delta variant 4 months after booster (116 days) displaying a mild form of disease. Other 8 subjects were infected with Omicron variant in the time frame post-booster 4.5–6 months (127–174 days, medium 147 days = 4.90 months) displaying very mild forms of disease (minor sympthoms for 1–3 days).

## Discussion

Testing the humoral response in COVID-19 and further in vaccination^37^, is important to correctly evaluate the immune response to the natural and/or artificial immunization^38^. Besides their privileged scientific value, these assesements were acutely demanded by the actual pandemic framework, when the whole flow of research, production, authorizing and use of the vaccines against COVID-19 was carried out at a rapid pace. Screening of vaccinated individuals represents a valuable tool for unravel the type and duration of the protective immune response, and for estimating the necessity of a booster dose, helping thus the health decision authorities in implementing important actions for controlling the pandemic and improve the vaccination scheme^39^. At least for a while, screening humoral immune response in vaccinated and infected people is very important to assess persistence of immunity in COVID-19, and evaluate the protective power of the SARS-CoV-2 antibodies^40^. In particular, serological testing could best catch the time-point when protective antibody levels begin to decrease and therefore this test should be included into vaccine effectiveness studies^39^. Thus, within the tested methods, the ELISA-related methodologies remain the most reliable and sensitive ones to evaluate the appeareance of specific antibodies^41^.

Adverse reactions to booster vaccination are reported as mainly pain at the injection site^42^, similar findings with our study. The adverse reactions panel reported in HCW receiving the first round of vaccination with BNT162b2 vaccine were significantly more frequent among HCWs with prior infection compared to infection-naïve individuals, and probably this process was due to the pre-existing cellular immunity. For the secound round of vaccination the total adverse reactions were milder^43^ thus this finding can reduce the overall negative attitude towards vaccines and vaccination.

A study performed on HCW in Greece has shown that the immune response after BNT162b2 vaccination dependents on sex and age^44^. We did not find statistically differences between the the antibody response in correlation with gender, age, or the registered co-morbidies, therefore additional studies can clarify these dependencies.

The strategy to follow a 3rd vaccination shows that priority should be given to high-risk groups, elderly and immunodeficiency patients. Numerous studies have shown that heterologous boosters inflict a higher immune response in comparison to homologous vaccination^45^. Therefore, the COV-ADAPT study has presented the results obtained in HCW receiving various vaccination protocols. Homologous ChAdOx1 nCoV-19, homologous BNT162b2 or heterologous ChAdOx1nCoV-19/BNT162b2 vaccinations protocols have induced different Spike protein-directed humoral and cellular immune responses^46^. An Israeli study has shown that BNT162b2, homologous booster dose was associated with a lower rate infection rate^47^. Our results show even after the first round of vaccination a reduced infection rate in our group and a low infection rate in the post-booster time frame. Although the post-booster infection was documented in almost 9% of the subjects, their symptoms were mild and the recovery was quick with no sequelae.

In Thailand, HCW receiving a third dose of AZD1222 were proved to trigger higher levels of specific IgG and IgA in comparison to the subjects receiving just two-dose vaccines. Moreover, higher neutralizing potency against the wild type and variants of concern were found in the group receiving the 3rd dose of vaccine^48^. We have obtained higher levels of IgG in the entire group after booster compared to the levels obtained after the first round of vaccination, while the IgA levels were statistically similar, our study confirming thus an earlier report^48^. Moreover, our results are in accordance to the study performed in Germany in HCW subjects. Thus, in the study it was shown that SARS-CoV-2-specific IgM and IgA decrease rapidly over time, whereas IgG decreases more slowly. Prior infected subjects induced after booster vaccination higher IgG levels and to a lesser degree IgA levels^49^. The link between the total antibodies and their neutralizing capacity is a question that still needs answears. A recent study, 2022, has shown that neutralizing titers are significantly higher post-boost compared to the titers obtained post two-dose series, as high as 15-fold increase in the neutralization capacity against Omicron variant. The mRNA booster dose induces an increase in both quantity and quality of the generated antibodies compared to the two-dose regimen^17^. Moreover, in a Finish study after booster vaccination, HCW group displayed similar with our study a high IgG concentrations and neutralizing antibodies were active against all variants, including Beta and Omicron variants^50^.

Re-infection after natural or artificial immunization after the booster shows that around 9% of our group showed documented respiratory infection, results that are in accordance with prior studies^51^ pointing out that genetically distinct new variants can avoid established immune memory.

## Study limitations

Comprehension of immune memory against SARS-CoV-2 viruses and their variants is still unknown. In general, studies show a 4, 6, 8 months waning of specific antibodies. Although this antibody wanning appears, tests on immune memory cells could perfectly complete the immune pattern of this respiratory infection. Our study performed on 103 HCW subjects may be considered as small, but the subjects were and still are throughly documented during these 2 years of pandemics. There are similar studies performed on small well documented groups. A similar study performed on 90 HCW subjects has shown that the median IgGs titers are decreasing monthly in both previously infected individuals and naive subjects. Seven months after vaccination, it was shown a dramatically decrease of the humoral response in all subjects^52^. Another study performed on 63 HCWs in Spain has shown that 2 months post-vaccination, antibody levels were decreased in naïve HCWs in comparison to previously infected HCWs. The authors report that 10 months post-infection, the immune system has an immunological memory capable of producing a rapid and powerful secondary antibody response^53^. In several cases that were weekly investigated post-booster we have shown that after vaccination, IgG level quickly increases, followed by a weekly increase of the IgA levels; this dynamic proving the clear existance of an immunological memory established by the first round of vaccination. The lack of correlation between the antibody response and the gender of the subjects can be explained by the fact that our group consisted of mainly females. We can not rule out that a more sex ratio group could have provided some correlations regarding geneder differences in the post-vaccination humoral response.

## Conclusion

Our results support the vaccination campaigns in highly exposed to infection professional healthcare workers receiving a booster dose of vaccine 8 months after the primary vaccination cycle. The administration of a third dose of mRNA vaccine as a booster addresses the potential waning of immunity over time and by-passes the inneficacy against future viral variants. Though, more information and clinical studies are required to verify the safety of heterologous vaccination strategies and the evaluation of the neccessity of a third dose of the vaccine. Although, our data show that there is a diminishing of the immune protection after 5 months after booster, the findings are opening the discussions for the need of an additional dose.

Within the vaccination or booster routine for a pandemic that is still on-going with its multiple waves and new variants, populational immunity remains an important issue for future implementation of prevention measures and control of this viral infection.

## Data Availability

The dataset presented in this study is available from the corresponding authors upon reasonable request.
